# Telocytes and interstitial cells of Cajal in the biliary system

**DOI:** 10.1111/jcmm.13643

**Published:** 2018-04-26

**Authors:** Lei Chen, Baoping Yu

**Affiliations:** ^1^ Department of Gastroenterology Renmin Hospital of Wuhan University Wuhan China; ^2^ Hubei Key Laboratory of Digestive System Disease Wuhan China

**Keywords:** biliary system, extrahepatic bile ducts, gallbladder, interstitial cells of Cajal, telocytes

## Abstract

A novel type of interstitial tissue cells in the biliary tree termed telocytes (TCs), formerly known as interstitial Cajal‐like cells (ICLCs), exhibits very particular features which unequivocally distinguish these cells from interstitial cells of Cajal (ICCs) and other interstitial cell types. Current research substantiates the existence of TCs and ICCs in the biliary system (gallbladder, extrahepatic bile duct, cystic duct, common bile duct and sphincter of Oddi). Here, we review the distribution, morphology and ultrastructure of TCs and ICCs in the biliary tree, with emphasis on their presumptive roles in physiological and pathophysiological processes.

## 1 INTRODUCTION

Using silver‐impregnation and methylene blue staining method, Ramon y Cajal discovered a particular cell type in the intestine of guinea pig and rabbit more than a century ago, when researchers regarded them as gut neurons.^1^ Approximately fifty years later, these cells, formerly called “interstitial neurons,” were rediscovered and renamed “interstitial cells of Cajal” (ICCs), according to contemporary electron microscopy examination.^2^, ^3^ In the gastrointestinal tract, ICCs initiate pacemaker activity and mediate neural inputs from the enteric nerve terminals to smooth muscles. They have a significant impact on the regulation of the spontaneous rhythmic activities of the gastrointestinal smooth muscle cells (SMCs).^3^, ^4^, ^5^, ^6^, ^7^, ^8^, ^9^ At the first decade of the 21st century, L.M. Popescu's group and other research teams detected interstitial Cajal‐like cells (ICLCs) in various tissues outside of the gut, including pancreas,^10^, ^11^ the muscle of atria and ventricles,^12^, ^13^ mammary gland,^14^ male and female genitourinary organs,^15^, ^16^, ^17^, ^18^, ^19^ mesentery^20^ and placenta.^21^ The group gave them this name in order to stress their apparent similarity with the canonical gastrointestinal ICCs. After a few years, in 2008, M.S. Faussone‐Pellegrini and her team observed ICLCs in the muscle coat of human gut and demonstrated they consistently differ from the ICCs both from ultrastructural and from immunophenotypic angles.^22^ To avoid further confusion and to give a precise identity to these cells, in 2010, Popescu and Faussone‐Pellegrini coined the term “telocyte” (TC) for them, on the basis of their unique and unequivocal features: the existence of a variable number (one to five) of cell body prolongations that are extremely long (tens to hundreds of μm, as measured on transmission electron microscope images), thin (<0.2 μm) and with a moniliform aspect.^23^ From then on, this novel cell type became known as the TC.^22^ In this review, we want to summarize current researches on ICCs and TCs in the biliary system and hence help to understand their underlying effect in health and disease.

## 2 ICCS IN THE BILIARY SYSTEM

Initially, ICCs have not been found in the gallbladder. Later on, Sun et al in 2006 found ICCs in the mice gallbladder, and in humans, Ortiz‐Hidalgo et al found in stromal tumour of the gallbladder cells with phenotype of ICCs.^24^ Early work substantiated that ICCs or TCs express CD117/Kit protein as a specific maker.^25^ Thus, Sun et al used immunohistochemistry employing a polyclonal antibody raised against CD117/Kit to confirm that c‐Kit was expressed in the gallbladder tissues of CD1 mice.^26^, ^27^ Spindle‐shaped ICCs with thin and long processes starting from the two poles appearing as a network‐like structure were found to be distributed in all the layers of the gallbladder wall. At the ultrastructural level, ICCs in the murine gallbladder have been reported to possess a well‐developed perinuclear endoplasmic reticulum, abundant mitochondria, free ribosomes, intermediate filaments and distinctive caveolae.^26^ These cells are adjacent to the smooth muscle cells and nerve endings.^27^ In addition, Ahmadi et al^28^ showed that ICCs are present in the human extrahepatic bile ducts, where they are more densely aggregated than those present in the gallbladder. These cells are most prominent within the muscle layers of the extrahepatic bile ducts where they are organized into loosely arranged laminae running parallel to circular muscle fibres. However, ICCs are invisible in intrahepatic bile ducts. The authors further suggested that the distribution and density of ICCs in the common bile duct are similar to those in the ampullary region, where they are distributed within the circular muscle, running mainly parallel to the circular direction, and are typically clustered in groups forming a loose network. However, the authors did not describe the distribution of ICCs in the longitudinal muscle layer of the common bile duct.^28^

There is now overwhelming evidence to support canonical ICCs as the origin of pacemaker potentials in the gastrointestinal tract, playing an important role in contractile activity. The gallbladder smooth muscle, which resembles the gastrointestinal smooth muscle, also exhibits spontaneous rhythmic electrical activity. Therefore, it has been proposed that in certain specific regions of the biliary system, these ICCs are likely involved in modulating the excitability of smooth muscle and tissue motility. This hypothesis was subsequently confirmed by researches conducted in China and abroad. In 2012, Pan et al^29^ explored the changes in gallbladder smooth muscle contraction and ICCs in the gallbladder using common bile duct ligation (CBDL) model in guinea pigs, and they found that both the contraction response of the gallbladder strips and the number of c‐Kit‐positive cells were reduced. The phenomena could be improved after relieving CBD obstruction. In recent years, our laboratory has dedicated effort to studying the correlation between ICCs and gallbladder motility in acute cholecystitis, too. For example, Huang et al^30^ reported that the expression levels of c‐Kit and SCF mRNA and protein were markedly decreased after CBDL. Accordingly, they proposed that the loss of ICCs may be attributed to repression of the SCF/c‐kit pathway during acute cholecystitis, which possibly participate in the development of decreased gallbladder motility. Moreover, as early as 2014, our colleague Zhang^31^ performed a similar experiment and observed that after ligation of the CBD, the gallbladder ICCs became swollen, had a dilated endoplasmic reticulum and a reduced number of cell processes. With prolonged time after CBDL, the injury to the gallbladder ICCs was further aggravated. At 48 hour after CBDL, the cytoplasm of the neutrophils was full of granules, and the neutrophils were in close contact with the ICCs bodies as well as their processes. Of particular note, the ultrastructural impairment of ICCs was slightly alleviated, and the cells moved closer to each other or to the gallbladder smooth muscle cells after using antipolymorphonuclear antibody (anti‐PNM). In addition, some authors recommended that ICCs are likely associated with choledochal cysts (CC). For instance, Duan^32^ suggested an involvement of the ICCs, which showed a significantly decreased density in the CC containing sparse ICCs remnants so that their network disappeared. This finding was compatible with reports by Zhao^33^ and Osman^34^ who proposed that reduction in the ICC population may contribute to the development of CC, and could help to disclose the abnormal motility of the common bile duct. However, CC represents an extremely rare congenital disorder that primarily manifests in the paediatric stage, characterized by intra‐ and/or extrahepatic biliary dilatation, although the exact aetiology remains incompletely understood.

## 3 ERA OF TCS IN THE BILIARY SYSTEM

In 2007, Lavoie et al^35^ identified the presence of TCs in the gallbladder of guinea pig. Around the same time, in human gallbladder, Hinescu et al^36^ also found these particular interstitial cells. Telocytes have been shown to differ from ICCs and all other interstitial cells, such as fibroblasts, fibrocytes, fibroblast‐like cells and mesenchymal stem cells. Telopodes (Tps), as are called the TC cellular extensions, are considered to be the longest cellular prolongation in the human body and the shape of the TCs is determined by the number of their Tps: piriform for one extension, spindle for two prolongations, triangular for three Tps and stellate for many extensions. Tps are usually described as having a moniliform appearance with podomers (thin, fibrillar segments) and podoms (dilated, cistern‐like regions), the latter of which contain mitochondria, endoplasmic reticulum and caveolae and are probably related to calcium uptake/release.^23^, ^37^

### 3.1 TCS IN THE GALLBLADDER

#### 3.1.1 The distribution and morphology of TCs in the gallbladder

Huang et al^38^ used conventional immunohistochemistry to detect TCs in the gallbladder of guinea pig and found that Kit‐positive interstitial cells having pleiomorphic and/or spindle‐shape and few bipolar processes were located in the smooth muscle layers. Notably, the number of TCs showed a gradual increase from the fundus to the neck of the gallbladder but the morphology was similar. Earlier, Lavoie et al^39^ investigated the distribution and characteristics of TCs in the gallbladders of the guinea pigs using morphological and physiological approaches. The results revealed that TCs were scattered throughout the extent of the gallbladder and did not appear to form a global network. Kit‐positive TCs, although more abundant along muscle bundles and near major blood vessels, were located in the subepithelial connective tissue layer as well as in the muscularis propria. In both regions, TCs had elongated cell bodies with one to two long primary processes (>100 μm); secondary processes were sometimes observed. Another research also proved that TCs appeared as two subpopulations—intramuscular TCs were located between smooth muscle cells forming one muscle bundle, and interbundle TCs were located in the connective tissue among smooth muscle bundles. These TCs usually exist as single cell, sometimes in small clusters of 2‐3 cells without forming a network‐like structure.^40^ In human gallbladder, Hinescu et al^36^ documented the presence of TCs in foetal and adult non‐tumoral gallbladder interstitium. TCs are mainly located near small vessels, in the subepithelial region of lamina propria, and between the smooth muscle bundles of the tunica muscularis. These cells were characterized by a network‐like appearance and thin, very long and moniliform cell prolongations. Given that antibodies against mast tryptase (normally found within mastocytes) can be used to differentiate between mast cells and TCs, Pasternak et al^41^ applied an indirect double immunofluorescence method to identify TCs in the human gallbladder. With this technique, they identified TCs throughout the area comprising the gallbladder fundus, body and neck. These cells were visible almost exclusively in the muscular layer, where they paralleled the smooth muscle cells. Together, these animal models and clinical experiments indicate that the shape of TCs slightly varies but their distribution is almost identical among species. Nevertheless, the conclusion that TCs in the gallbladder form a cellular network in various species is controversial, which requires further study to resolve.

#### 3.1.2 Ultrastructure of TCs in the gallbladder

Transmission electron microscopy has revealed the characteristics of TCs in the gallbladder of the guinea pig as elongated, electron‐dense cells with multiple long interconnected processes that are rich in mitochondria, smooth endoplasmic reticulum and caveolae. TCs are intercalated between nerves and SMCs and form direct appositions and/or gap junctions with surrounding cells.^39^ Hinescu et al^36^ provided a detailed description of the ultrastructural features of TCs in the human bladder: (1) location in the non‐epithelial space; (2) close contacts with targets: nerve bundles, and/or epithelial cells, and/or SMCs and/or capillaries; (3) characteristic cytoplasmic processes: (i) numbers (1‐3, frequently: 2‐3); (ii) length (tens up to hundreds of μm); (iii) thickness (uneven calibre, <0.5 μm); (iv) aspect: moniliform, usually with mitochondria in dilations; (v) presence of “Ca^2+^ release units”; (vi) branching: dichotomous pattern; (vii) organization in network—labyrinthic system: overlapping cytoplasmic processes; (4) gap junctions: with SMCs or with each other; (5) basal lamina: occasionally present; (6) caveolae: 2%‐3% of cytoplasmic volume; ~0.5 caveolae/μm of cell membrane length; (7) mitochondria: 5%‐10% of cytoplasmic volume; (8) endoplasmic reticulum: about 1%‐2%, either smooth or rough; (9) cytoskeleton: intermediate and thin filaments, as well as microtubules, present; (10) myosin thick filaments: undetectable. The ultrastructure of TC in the gallbladder is quite similar among humans and other mammals.

#### 3.1.3 The possible role of TCs in the gallbladder

The excitation‐contraction coupling that is well known to occur in the gastrointestinal smooth muscle is also present in the gallbladder smooth muscle (GBSM) cells. However, GBSM cells are arranged as interdigitated bundles orientated in multiple directions, which is in contrast with the sheet arrangement of smooth muscle cells observed in the gastrointestinal tract.^42^, ^43^ Through electrocholecystogram analysis, Shafik^44^ demonstrated that the gallbladder generates an electrical activity in the form of pacesetter potentials (PPs) and action potentials (APs), and the APs follow the PPs randomly. It is likely that the electrical waves start at the gallbladder fundus and proceed towards the cystic duct. Rhythmic excitation in the gallbladder is multifocal among smooth muscle bundles and can be synchronized by excitatory agonists;^43^ however, it remains unclear as to precisely how GBSM cells generate and propagate such rhythmic excitation. Intercellular Ca^2+^ transients indicate Ca^2+^ influx via L‐type Ca^2+^ channels during action potentials, which were detected in the guinea pig gallbladder SMCs and TCs as rapidly occurring Ca^2+^ flashes.^39^ Lavoie et al^39^ observed that spontaneous rhythmic activity and Ca^2+^ flashes in smooth muscle cells were synchronized with the electric activity and Ca^2+^ transients of TCs in a given bundle and that Ca^2+^ flashes in TCs exhibited higher intensity and longer duration when compared to gallbladder SMCs. They also found that gap junction uncouplers eliminated or greatly reduced Ca^2+^ flashes in gallbladder SMCs, but the rhythmic activity persisted in TCs, whereas treatment with a Kit tyrosine kinase inhibitor eliminated or reduced APs and Ca^2+^ flashes in both cell types, as well as associated tissue contractions. These results pointed to the existence of electrical coupling between TCs and gallbladder SMCs, suggesting that TCs may be involved in the generation and propagation of the spontaneous rhythmicity of gallbladder tunica muscularis. This speculation was further substantiated in a recent study showing that the amplitude and frequency of the slow wave were remarkably reduced; concurrently, the tonic contraction was significantly declined in gallbladder smooth muscle strips with impaired TCs.^40^ Moreover, Balemba et al^45^ demonstrated that spontaneous activity in gallbladder TCs was abolished by treatment with mitochondrial inhibitors, emphasizing the essential role of mitochondrial Ca^2+^ handling in generating spontaneous rhythmicity of TCs and regulating excitability of gallbladder. Overall, these observations support a role of TCs in the generation and propagation of pacemaker activity and in the regulation of gallbladder motility.

#### 3.1.4 Distribution of receptors on TCs in the gallbladder

A characteristic feature of interstitial TCs in the gallbladder is expression of the transmembrane tyrosine kinase receptor c‐Kit, which enables their convenient identification by immunohistochemical and molecular methods. However, gallbladder mastocytes are also immunopositive for CD117/Kit receptor tyrosine kinase. It is generally accepted that application of antibodies against mast tryptase or toluidine blue staining can effectively distinguish between mast cells and telocytes. Pasternak^46^ found that tryptase immunolabelling could help to identify more mastocytes than toluidine blue staining since even the partially degranulated cells remained tryptase‐immunopositive. In immunostained slides, the c‐Kit and tryptase double‐positive mast cells were generally round‐ or oval‐shaped, had a centrally located nucleus and were predominantly localized in the lamina propria than in the muscularis propria. Based on these observations, TCs can be defined as c‐Kit‐positive nucleated cells that lack mast cell tryptase expression.

Cholecystokinin is released from mucosal endocrine cells in the proximal small intestine in response to a meal, and it is classically known to stimulate the gallbladder contraction. In an early study, researchers identified cholecystokinin A receptor (CCK‐AR) expressed on gallbladder ICCs of guinea pig.^47^ Of note, in 2015, Fan et al^40^ suggested that CCK‐evoked contraction may be partially mediated through the direct action of CCK‐AR on gallbladder TCs. After incubation of gallbladders with methylene blue and subsequent intense illumination, the TCs were shown to be clearly swollen while the SMCs were not affected. They further observed that cholecystokinin–octapeptide (CCK‐8) treatment results in a significant right‐shift of the contraction dose‐response curve.

### 3.2 TCS IN THE EXTRAHEPATIC BILE DUCT (CYSTIC DUCT, THE COMMON BILE DUCT, THE SPHINCTER OF ODDI)

Lavoie et al^39^ demonstrated elongated TCs in the hepatic and cystic ducts of guinea pig. Likewise, Huang et al^38^ found TCs in extrahepatic duct and cystic duct, where these cells, with one or two processes, were usually dispersed in the muscular bundles. Additionally, Huang et al^38^ proposed that in the common bile duct, the density and number of TCs are gradually increased from the upper to the lower part along with thickening of the smooth muscle layers. However, it has also been noted that the density of TCs is rapidly reduced within 1‐2 mm of the distal end of the common bile duct.^39^ In the upper portion of the common bile duct, TCs are located in the muscular bundles orientated parallel to the circular muscle and have oval‐shaped cell bodies measuring about 7.2‐9.4 μm in diameter and bipolar processes of approximately 50‐100 μm in length. Circular muscle coat gets thicker and longitudinal muscle bundles begin to appear in the middle part of the common bile duct, where TCs with extended processes are arranged in both circular and longitudinal directions. At the lower portion of the common bile duct, numerous TCs with few processes form a complicated network between the circular and longitudinal smooth muscle layers.

The aboral side of the common bile duct, where the ampulla opens into the duodenum, is surrounded by the sphincter of Oddi (SO). It has a rather complex muscular structure because the SMCs mainly run in a circular direction along with several longitudinal or oblique muscle bundles. Within the sphincter of Oddi, Huang et al^38^ showed that the Kit‐positive TCs were located in the smooth muscle layers (bundles). Most of them were spindle‐like in shape with 2‐3 processes of 150‐250 μm in length; some of them had oval cell bodies, about 7‐9 μm in diameter, with short branches. Moreover, a few TCs had 3‐5 processes with few branches measuring about 100 μm in length. These TCs lay parallel to the SMCs and formed a cellular network.^38^ Another investigator, Lavoie et al^39^ demonstrated in the sphincter of Oddi, that telocytes, having elongated bodies with multiple processes, were located in the circular and longitudinal muscle layers and arranged parallel with them. Those cells formed a network and were found in the ganglionated myenteric nerve plexus area, overlying the nerve plexus,^39^ in agreement with those results obtained in other studies. Moreover, analysis of the spontaneous rhythmic myoelectric activities in the sphincter of Oddi was confirmed to match this distribution and morphology of TCs.^48^ In addition, telocytes and their processes are usually found adjacent to the nitric oxide synthase‐positive neurons in the sphincter of Oddi,^49^ suggesting that TCs may act as the target cells of the neurotransmitter NO, contributing to regulation of the spontaneous rhythmic electrical activities and development of motility disorders of the sphincter of Oddi.

## 4 TCS AND BILIARY SYSTEMIC DISEASE

Since the discovery of TCs in the biliary tree, increasing evidence suggests a correlation between these cells and biliary systemic motility as highlighted above. Accordingly, their potential role in the pathogenesis of some biliary systemic disorders has now begun to attract substantial attention.

### 4.1 TCs and cholelithiasis

Cholelithiasis is one of the most common diseases in gastroenterology that is caused by concrement in the biliary system (ie gallbladder, extra‐ and intrahepatic bile duct). Several factors are involved in the pathogenesis of this disease, including cholesterol hypersecretion with supersaturation bile, gallbladder hypomotility, as well as accelerated cholesterol crystallization in the bile. The cholesterol saturation index (CSI) is an accepted parameter relevant to bile lithogenicity.^50^ Moreover, it is known that smooth muscle, enteric nervous system and the recently described telocytes are responsible for the regulatory mechanisms of gallbladder motility. In a recent study, Pasternak et al^51^ compared the lipid contents of bile samples from patients with and without cholelithiasis and showed that the cholelithiasis group had a statistically significantly higher CSI, which exhibited a negative correlation with reduced TCs in the gallbladder wall. This indicates that the bile in patients with gallstones is highly lithogenic, which may result in impaired gallbladder motility. Briefly, cholesterol accumulation in the gallbladder smooth muscle cells destroys the signal transduction mediated by protein G resulting from CCK‐A binding to its receptor.^52^ On the other hand, an excess of cholesterol in the membrane of the caveolae probably reduces membrane fluidity,^53^ further leading to disturbances in cytosolic calcium homeostasis. Such disturbances of membrane and cytosolic calcium homeostasis would result in disorder of the membrane potential oscillation in TCs, because abundant Ca2+ is required to generate spontaneous electrical activity, thus affecting the pacemaker function of these cells. In another study, Pasternak et al^54^ further evaluated the relationship between the biliary lipid composition and TCs density in the gallbladder wall of patients with gallstone and found that the ω‐6 polyunsaturated fatty acid (PUFA) concentrations were significantly elevated, whereas the mean concentrations of glycocholic acid (GCA) and taurocholic acid (TCA) in the bile were markedly reduced. A high level of ω‐6 PUFA was shown to contribute to cholelithic formation by enhancing biliary cholesterol secretion,^55^ whereas GCA and TCA may have a protective effect on TCs based on a significant positive correlation between the mean number of TCs and the concentrations of GCAs and TCAs in patients with gallstone.^54^ Therefore, these observations support that the higher concentration of ω‐6 PUFA and the lower amounts of GCA and TCA result in TCs loss and consequent gallstone formation by elevating the lithogenicity index.^54^ Moreover, some authors suggested that damage to TCs could be related to the blocking of the c‐kit/SCF signal pathway consequent to a high cholesterol level^40^ or chronic inflammatory reaction in the gallbladder wall.^46^ In conclusion, an increased bile lithogenicity index or other factors cause the deficiency of TCs, which may further induce the functional impairment of gallbladder motility, particularly given the importance of TCs in gallbladder rhythmic electrical activity.

### 4.2 TCs and cholecystitis

In our most recent study,^56^ we explored the effects of neutrophils on TCs in the gallbladder and examined the possibility that TCs take part in gallbladder hypomotility during acute acalculous cholecystitis induced by CBDL. The result revealed damage to the ultrastructure of TCs in both co‐culture 2 group (TCs co‐culture with neutrophils isolated from 24‐hours CBDL group) and co‐culture 3 group (TCs co‐culture with neutrophils isolated from 48‐hours CBDL group). In addition, the co‐culture groups showed a significantly higher number of apoptotic TCs than the group of culture alone, and co‐culture 2/3 group was higher than co‐culture 1 group (TCs co‐culture with neutrophils isolated from the sham‐operated group). What's more, the protein and mRNA levels of both SCF and c‐kit decreased in all three co‐culture groups, and the lowest expression level was detected in co‐culture 3 group. All above results suggested that the decreased gallbladder motility in acute cholecystitis is due to the effects of neutrophils on the development and function of gall bladder TCs via depression of SCF/c‐kit expression. But the specific mechanisms underlying this process remain to be further illuminated.

### 4.3 TCs and stromal tumours in the biliary tree

Gastrointestinal stromal tumours (GISTs) are the most common primary, non‐epithelial, mesenchymal tumours of the tubular GI tract, which originate from or manifest the morphological and immunophenotypic characteristics of ICCs. Gradually, stromal tumours which are similar in immunophenotype to TCs [CD117 (c‐Kit)+/CD34+] have also been sporadically discovered outside of the tubular gut, including pancreas, liver, and gallbladder, which are named as extragastrointestinal stromal tumours (EGISTs). In a recent study, Padhi et al^25^ summarized that six of nine EGISTs in the gallbladder were associated with gallstones, and these stromal tumours showed typical spindle‐shaped cells that were consistently immunoreactive for CD117/c‐Kit as well as CD34. In addition, Ortiz‐Hidalgo et al^57^ suggested that the stromal tumour of the gallbladder results from non‐physiological hyperplasia of gallbladder ICCs. While Furihata et al^58^ reported a case of GIST‐resembling, malignant stromal tumour arising in the gallbladder, based on microscopy and immunohistochemistry, which was differentiated into striated muscle. Given these findings, the origin of EGISTs and the exact pathophysiologic effect in the biliary tree need to be further elucidated.

## 5 SUMMARY

Telocytes and ICCs have been identified in the biliary tree, including gallbladder, cystic duct, extrahepatic bile duct, common bile duct and sphincter of Oddi. TCs in the biliary system are similar to canonical enteric ICCs in the aspect of morphological characteristics and ultrastructure, and they may have a functional relationship with adjacent SMCs and enteric neurons. TCs can be generally divided into two classes according to their location in the biliary tract, namely subepithelial and intramuscular cells. However, it is still not clear whether TCs in the gallbladder form a cellular network. As gallbladder and sphincter of Oddi exhibit spontaneous rhythmic contraction, it is likely that TCs and ICCs regulate excitability of SMCs and tissue motility in specific areas by modulating the generation and propagation of electrical activity. Moreover, there is ample evidence that TCs and ICCs in the bile tree may be related to the development of cholelithiasis, acute cholecystitis, choledochal cysts and stromal tumours of the gallbladder. Works performed in order to understand the role of gallbladder TCs and ICCs under physiological condition has provided new insights and exciting new hypotheses about the causes of some biliary systemic disorders, which can help to give rise to novel potential therapies for these diseases. Nevertheless, the current knowledge pertaining to TCs and ICCs in the biliary tree remains extremely limited, and thus, more studies are certainly needed in both normal and diseased biliary system to advance this field.

## CONFLICTS OF INTEREST

The authors confirm that there are no conflicts of interest.
